# 

**Published:** 1889-11-02

**Authors:** 


					1 '
SJThomas's Hospital
Clascok Western Infirmary
1RWICH HOSPl TAL
IORFOLKaub
SPECIAL MEDICAL NUMBER
No. 162, Vol. VII.]
Saturday, November 2, 1889.
[One Penny.

				

